# Supplementary material for: Causal Relationship between Aging and Anorexia Nervosa: A White-Matter-Microstructure-Mediated Mendelian Randomization Analysis
Source: Biomedicines. 2024 Aug 16;12(8):1874. doi: 10.3390/biomedicines12081874 (PMC11351342; doi:10.3390/biomedicines12081874)
Supplement: Supplementary file 1 [file biomedicines-12-01874-s001.zip › Supplementary Figure/Figure S8/Leaveoneout plot of MR analysis for TL to ubm-b-2091.pdf]

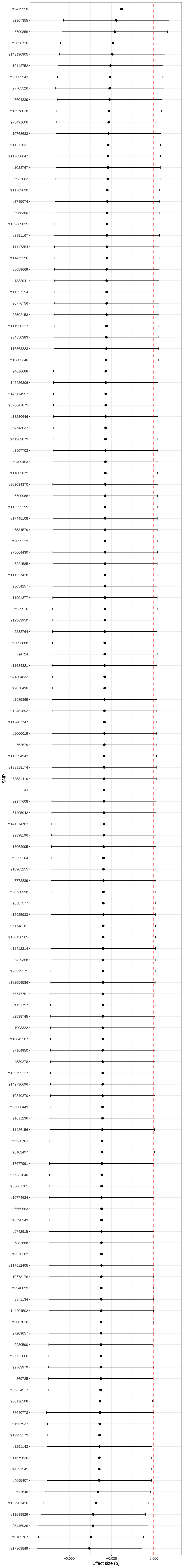
rs9419958
rs2967355
rs7790856
rs2056726
rs143190905
rs10112752
rs76065543
rs7705526
rs45604339
rs16978028
rs78491606
rs10768683
rs11212631
rs117630647
rs1023767
rs932002
rs11769630
rs3785074
rs9955360
rs139669835
rs3891167
rs11117354
rs11412296
rs6659669
rs1332941
rs11557154
rs6776756
rs28502153
rs111950327
rs34550383
rs116863223
rs10805346
rs4616688
rs142426306
rs145114957
rs376641875
rs13230646
rs4743037
rs41269079
rs1907702
rs59409453
rs11085072
rs202034370
rs6790988
rs113525195
rs17445108
rs6584579
rs7099229
rs75664430
rs7221585
rs111527438
rs6054257
rs11991877
rs939916
rs12369950
rs2282764
rs3093888
rs4724
rs11584821
rs41304832
rs9878436
rs1985369
rs12451892
rs117407747
rs9600019
rs762678
rs112394943
rs188918174
rs73581419
A8
rs2977608
rs61405042
rs141214782
rs9398196
rs13062095
rs2555104
rs10905255
rs7772289
rs73730598
rs6587577
rs12925933
rs61748181
rs150150565
rs12412214
rs429358
rs76219171
rs182059586
rs55747751
rs131797
rs2538745
rs1003322
rs10845387
rs7164950
rs4530278
rs139795227
rs142730696
rs10840270
rs76666449
rs1611236
rs11426156
rs6536702
rs8102497
rs17677991
rs77231040
rs56061761
rs10774624
rs6669563
rs6590343
rs5742915
rs6881568
rs2276182
rs117512405
rs10773176
rs9940099
rs871134
rs144204502
rs6007020
rs7209057
rs2230590
rs77732866
rs2763979
rs869785
rs80324517
rs80116508
rs35640778
rs1957937
rs12932179
rs1291143
rs11579626
rs4731541
rs4695407
rs611646
rs137901416
rs11699829
rs35446936
rs8105767
rs17803849
SNP
-0.050
-0.025
0.000
Effect size (b)
